# Exploring nursing students’ experiences of using generative AI for emotional intelligence learning: a qualitative study

**DOI:** 10.3389/fpubh.2026.1967799

**Published:** 2026-10-12

**Authors:** Xiaohui Fan, Jingjing Sun, Rui Xu, Linghui Zhang, Ruixiao Ou, Ruihua Jin, Hui Yang, Panpan Huai

**Affiliations:** 1 Biomedical Research Center, School of Medicine, Xijing University, Xi’an, China; 2 School of Nursing, Shanxi Medical University, Taiyuan, China; 3 School of Nursing, Shanxi Technology and Business University, Taiyuan, China; 4 First Hospital of Shanxi Medical University, Taiyuan, China

**Keywords:** emotional intelligence, generative artificial intelligence, qualitative research, TPACK framework, undergraduate nursing students, UTAUT model

## Abstract

**Background:**

Emotional intelligence is a core competency for nursing students; however, traditional nursing education faces challenges in fostering emotional intelligence, including curricular imbalances, limited teaching methods, and delayed assessment and feedback. The rapid development of generative artificial intelligence (GenAI) offers new possibilities for addressing the above issues; however, existing research has not yet thoroughly explored how nursing students learn emotional intelligence through GenAI or their experiences in this process.

**Objective:**

Based on the Unified Theory of Acceptance and Use of Technology (UTAUT) and the Teaching Pedagogical Content Knowledge (TPACK) framework, and using Goleman’s five-dimensional model of emotional intelligence as a reference, this study explores the experiences of undergraduate nursing students in using generative artificial intelligence for emotional intelligence learning, as well as the factors that facilitate this process.

**Method:**

This study employed a qualitative descriptive research design and Reflexive Thematic Analysis (RTA). Using purposive sampling, 19 undergraduate nursing students (from freshman to senior year) were selected for one-on-one semi-structured in-depth interviews. All participants had experience using GenAI tools (such as DeepSeek and Doubao) for communication practice, case discussions, or sentiment analysis. The interview data were analyzed using the RTA six-stage process, with NVivo 12.0 software used to assist with data management.

**Results:**

We identified and synthesized 238 initial codes into three core themes. Theme 1, “From Efficiency-Driven to Precise Empathy,” presents the differing descriptions of empathy-related experiences among students in different grade levels: lower-grade students more frequently reported efficiency-driven usage experiences; some middle-grade students described difficulties caused by technical barriers; and upper-grade students more often described the recognition of patients’ emotions in conjunction with clinical experience. Theme 2, “From Mimicking AI to Autonomous Expression,” focuses on students’ reported experiences related to social skills, including direct application, recognition and adjustment, autonomous expression, and the simulation gap that persisted throughout the process. Theme 3, “From Technical Frustration to Mental Rehearsal,” integrates experiences related to self-management, self-awareness, and self-motivation. The three themes correspond to different dimensions of emotional intelligence, with students in different grade levels reporting distinct experiential characteristics.

**Conclusion:**

Nursing undergraduates in different grades exhibit distinct experiential characteristics when using generative artificial intelligence to learn about emotional intelligence. Students’ reports repeatedly highlighted moderate tension around technological motivation, the ability to critically integrate technology, and the perceived value of technological friction. Based on these exploratory findings, we propose several preliminary educational implications—including grade-level-specific support, curriculum exploration, and education in critical technological literacy and ethics—for consideration in future research and practice.

## 1 Introduction

Emotional intelligence refers to the ability to recognize, understand, and manage one’s own and others’ emotions, as well as the ability to use emotional information to guide thinking and behavior (1). Goleman (1998) further emphasizes that emotional intelligence encompasses not only the recognition and regulation of emotions, but also an understanding of how emotions influence an individual’s perspectives and actions (2). Research indicates that in nursing practice, emotional intelligence has become a key personal resource for establishing therapeutic nurse–patient relationships, coping with occupational stress, and maintaining effective interpersonal relationships (3, 4). For nursing students, clinical rotations and academic studies involve navigating complex interpersonal interactions, including communication with patients, family members, and clinical instructors, as well as emotional fluctuations triggered by academic pressures and clinical situations. Nursing students with high emotional intelligence are better able to perceive others’ nonverbal emotional cues and demonstrate greater empathy and stress resilience, enabling them to make more prudent decisions in simulated or real-world clinical interactions (5). Therefore, emotional intelligence is not only a core competency for practicing nurses but also helps nursing students manage interpersonal relationships and interpret emotional cues during their education, laying a crucial foundation for their future professional success (6, 7).

GenAI, a major branch of artificial intelligence, refers to tools that take user-provided written prompts and generate output based on them (8). With the rapid development of generative artificial intelligence, existing research has begun to explore its potential to foster emotional intelligence among nursing students. These studies primarily examine individual cognitive mechanisms from a psychological perspective, such as the influence of technology anxiety and self-efficacy on willingness to use (9, 10); or employ cross-sectional designs to investigate students’ acceptance and satisfaction with AI tools (5, 7), providing preliminary evidence of the effectiveness of generative AI in enhancing communication and empathy (11, 12). However, there remains a lack of systematic and in-depth exploration of how students perceive and describe their experiences with artificial intelligence at the cognitive, affective, and behavioral levels, as well as the relationships among technology, learning content, and learning strategies during human-computer interaction (8, 13).

To systematically investigate how students adopt and utilise GenAI for emotional intelligence learning in practice, this study integrates the Unified Theory of Acceptance and Use of Technology (UTAUT) with the Technology-Pedagogical Content Knowledge (TPACK) framework. The Unified Theory of Acceptance and Use of Technology (UTAUT), proposed by Venkatesh et al. in 2003, is one of the most influential theoretical models in the field of information technology acceptance and is widely applied in research on user technology adoption behavior across sectors such as education, healthcare, and business (9). This theory analyzes the key factors influencing students’ acceptance and use of AI from the perspective of behavioral motivations (such as performance expectations, social influence, and facilitating conditions) (9); meanwhile, the Technology Pedagogical Content Knowledge (TPACK) framework, originally proposed by Mishra and Koehler in 2006, focuses primarily on the knowledge structure required for teachers to integrate technology (14). In 2009, they further noted that TPACK can also be applied to students, requiring teachers to understand “how technology helps students overcome learning difficulties, as well as students’ prior knowledge and epistemological theories” (15). In other words, the TPACK framework includes an analysis of students’ cognitive states, which provides a theoretical basis for its application in student research. From the perspective of knowledge integration, this theory explains how students organically combine technological knowledge (TK), content knowledge (CK) in nursing, and pedagogical knowledge (PK) to achieve effective instructional integration (14). Integrating these two frameworks helps us understand “why students use AI” (UTAUT focuses on behavioral intentions and acceptance) and “how students describe the integration of technology and knowledge” (TPACK focuses on knowledge integration and instructional integration), thereby providing a theoretical perspective for this study’s analysis of students’ descriptions of their experiences using GenAI (16). Specifically, UTAUT explains why students use AI (behavioral motivations), TPACK explains how students integrate technology and knowledge (process mechanisms), and the Goleman model provides a reference framework for the emotional intelligence dimension.

Therefore, this study aims to explore undergraduate nursing students’ perceptual experiences using generative artificial intelligence (GenAI) in both classroom and out-of-class settings, as well as the factors they report influencing these experiences. A previous scoping review by our research team found that existing strategies for cultivating emotional intelligence in medical students using GenAI primarily focus on empathy and social skills, while research on self-awareness, self-management abilities, and self-motivation mechanisms remains relatively limited. At the same time, existing research has largely focused on the immediate effects of GenAI tools, with little in-depth exploration of how students describe their experiences integrating GenAI tools, nursing knowledge, and emotional training strategies in specific professional coursework (17). Based on this, the core research questions this study seeks to address include: (1) How do undergraduate nursing students perceive the role of generative AI in their emotional intelligence learning? (2) What different experiences do students from different academic years describe when using generative AI for emotional intelligence learning? (3) What factors do students perceive as influencing their use of generative AI for emotional intelligence learning? How do they describe the relationship among technology, nursing knowledge, and communication strategies? By addressing these questions, this study aims to provide a solid empirical foundation and theoretical guidance for the scientific design and effective interventions in nursing emotional education in the era of artificial intelligence.

## 2 Methods

This study employed a qualitative descriptive design and utilized the Reflexive Thematic Analysis (RTA) method proposed by Braun and Clarke (2022) (18). RTA emphasizes treating the researcher’s subjectivity as a resource for analysis and identifies patterns of meaning in the data through a systematic six-step process. This report adheres to the Criteria for Reporting Qualitative Studies (COREQ) checklist (19) and the RTARG guidelines (18) to ensure transparency in data collection and analysis, as well as methodological rigor. This study employs a retrospective, self-report interview design aimed at exploring students’ perceived experiences and descriptions of the learning process, rather than objectively measuring changes in emotional intelligence through pre- and post-tests or behavioral assessments (20). The study focuses on how students describe and perceive their experiences using generative artificial intelligence for emotional intelligence learning, rather than verifying whether objective changes in emotional intelligence have occurred.

### 2.1 Design

A qualitative study exploring nursing undergraduates’ perceptions and experiences of using generative artificial intelligence for emotional intelligence learning through in-person, one-on-one, semi-structured interviews. To ensure coherence across interviews, the interview structure developed by Krueger and Casey (21) was adopted, as it includes questions for introductions, transitions, and key topics. An outline of the interview framework is presented in Table 1.

**Table 1:** Sample questions for semi-structured interviews.

Content	Example
Introduction	1. Could you briefly tell us about your major and year of study? What clinical placements or internships have you completed so far?
	2. In your opinion, what does a nurse’s “empathy” or “emotional sensitivity” mean in clinical practice? Why is it important?
Transiton issues	3. In your studies, aside from completing assignments, have you ever taken the initiative to use AI tools like DeepSeek or Doubao? What do you typically use them for? Please describe specifically which courses or assignments you have used them in.
Key issues	4. Think back to a time when you used AI to simulate the emotions of a patient or family member. How did you set up that role? Did you think the AI simulation was convincing?
	5. In that simulated conversation, what role did you assume? What communication techniques or strategies did you try? How does conversing with an AI differ fundamentally from role-playing with a classmate?
	6. Have you ever noticed that an AI’s response “did not quite feel right” during a conversation? How did you realize this and handle it at the time?
	7. Through this simulated interaction with AI, do you feel that you have become more attuned to the emotions conveyed in patients’ words? Could you give an example?
	8. Have the responses or suggestions provided by AI subtly influenced the way you speak when comforting patients, explaining medical conditions, or handling conflicts in real life? Do you apply them directly, or do you adapt them based on your own judgment? Can you describe a specific change?
	9. After repeatedly practicing how to handle a particularly challenging communication scenario using AI, how do you feel your emotional control and mindset have changed when you think about a real clinical setting?
	10. Have you ever used AI to analyze or process your emotions? Did the AI’s responses help you gain a clearer understanding of your emotions?
	11. While practicing emotional communication with AI, have you ever encountered situations that made you feel frustrated or angry? How did you manage your emotions?
	12. What factors motivate you to continue using AI to practice emotional communication? Does the attitude of those around you have a significant influence on you?
	13. Does it help you complete these types of tasks effectively? Is the process smooth? Have you ever found yourself unsure of how to “ask” it something?
	14. Overall, what kind of external support or guidance do you think is needed to make better use of AI for this type of emotional communication exercise?
End of the issues	15. Based on your experience, what do you think is the biggest shortcoming or potential risk of using AI for emotional communication training at this time?
	16. To what extent do you think the experience and confidence gained from AI simulations can be applied to real-world clinical settings? What might be the main obstacles to effectively putting this knowledge into practice?
	17. If the school hopes to systematically integrate these types of AI tools into its nursing curriculum in the future, what do you think are the key issues that need to be addressed?

### 2.2 Sample

The participants are enrolled in a four-year undergraduate nursing program at a university in northern China. The program’s curriculum consists of 3 years of theoretical instruction and clinical rotations, followed by a clinical internship in the fourth year. Clinical rotations are concentrated in the third year, while the clinical internship is scheduled for the fourth year. In addition, the program includes comprehensive nursing practical training courses, virtual simulation systems, and extracurricular clinical practice activities. It is worth noting that earlier cohorts of students in this program had limited exposure to artificial intelligence during their studies, whereas the Class of 2025 has been immersed in an educational environment marked by widespread digital transformation and the deep integration of artificial intelligence since their enrollment. This defining feature of the era has profoundly shaped their contextualized emotional learning experiences.

Purposive sampling was used to recruit students. Recruitment was halted during the data collection process once information saturation was reached, defined as the absence of new themes and the emergence of repetitive responses (22). In this study, we reached information saturation after the 16th interview and conducted three additional interviews to confirm that no new themes had emerged. The final sample size was 19 participants, which falls within the recommended range for interviews in a single qualitative study, as outlined in the methodological guidelines for reflexive thematic analysis (RTA) (18). The inclusion criteria are as follows: (1) full-time undergraduate nursing students; (2) prior experience using AI tools such as DEEPSEEK or Doubao; (3) use of GenAI tools to assist with communication exercises, case discussions, or emotion analysis; (4) voluntary participation in the interview and signing of an informed consent form.

### 2.3 Rigor, credibility, and reflectiveness

The rigor and credibility of this study are established based on the criteria proposed by Lincoln and Guba (23) and adhere to a four-dimensional framework comprising credibility, transferability, reliability, and verifiability (24). By conducting semi-structured interviews in a quiet, private setting, we ensured that participants had ample time to express their experiences and subjective feelings, thereby enhancing the credibility of the research findings. After thematic analysis was completed, the preliminarily identified themes and representative quotes were emailed to three participants for member validation to confirm that the interpretations aligned with their experiences. All participants confirmed the findings and did not propose any major revisions. Additionally, the research team held regular discussion meetings to collectively review the fit between the coding and the themes. To ensure generalizability, the study provided detailed contextual descriptions, including the research site’s curriculum, student grade-level characteristics, GenAI usage contexts, and sample diversity. A combination of maximum diversity sampling and snowball sampling was employed, ensuring that participants’ grade rankings spanned the top 10% and bottom 20% of the student body to enhance the transferability of the findings. Reliability was ensured through the use of a standardized interview guide for all participants, ongoing team discussions during the data analysis phase, a comprehensive review of findings, and the maintenance of a complete audit trail (including interview records, transcripts, NVivo coding files, analysis matrices, team meeting minutes, and researcher reflection logs). Verifiability was achieved through researchers’ reflexive practices: all researchers received specialized training in qualitative research methods prior to data collection, strictly maintained participant anonymity throughout the analysis process, and directly cited participants’ original statements in the research findings. Each researcher independently maintained a reflection journal documenting their personal attitudes toward GenAI, theoretical stances, and potential influences on data interpretation. The team regularly shared and discussed these journals and proactively examined individual biases to ensure that research findings authentically reflected participants’ responses rather than researchers’ subjective assumptions.

Our research team consists of three graduate students specializing in nursing education, all of whom hold nursing degrees and have received training in qualitative research methods and communication skills. The team’s faculty advisor is affiliated with the same institution and has extensive experience in writing and supervising qualitative research, having published numerous papers in the field’s journals.

### 2.4 Data collection

Data collection took place in March 2026 using purposive sampling, with undergraduate nursing students at a university in China selected as the study participants. At the beginning of the study, preliminary interviews were conducted with three participants to refine the interview guide. The interviews were conducted in a quiet, undisturbed environment. With mutual consent, the interviews were audio-recorded, subsequently transcribed verbatim, de-identified, and stored in a password-protected folder. During the interviews, the researcher listened attentively, took notes in real time, provided appropriate responses, and offered emotional support. Participants were free to withdraw from the interview at any time if they felt uncomfortable. Participants will receive a small gift as a token of appreciation for their participation in this study.

### 2.5 Ethical considerations

The research protocol was approved by the Institutional Review Board of a university in China (No. XJU202502). Participant privacy was protected. All participants provided written informed consent. No harm came to the participants. All nursing students had the right to choose whether to participate in this study. Data were securely stored and encrypted. Participants were free to withdraw at any time without any adverse effects on their studies or academic performance.

To ensure confidentiality, interviews were recorded only after obtaining informed consent. In accordance with the guidelines and ethical standards of relevant organisations, we will retain the data for 2 years after the study’s completion, and the corresponding author may request it during this period. After this period, the data will be permanently and securely deleted. Furthermore, to protect the privacy of students in the case studies, participants were explicitly instructed before the interviews to avoid disclosing any personally identifiable information. During data processing, participants were coded with random numbers ranging from N1 to N19, and any personal identifiers mentioned in the interviews were uniformly replaced with “X”.

### 2.6 Data analysis

All interviews were verbatim recorded and transcribed with the participants’ consent. Within 24 h of each interview, the researchers transcribed the audio recordings and cross-checked them with their field notes. Data analysis employed the Reflexive Thematic Analysis (RTA) method proposed by Braun and Clarke (2022) (18), following their six-stage process:

Data familiarization: the first author (SJJ) repeatedly read and reread the interview transcripts while listening to the audio recordings to deepen understanding of the data’s context and emotional tone. During this process, SJJ took preliminary reflective notes, paying particular attention to differences in expression patterns among students across grades and to narrative segments related to the development of emotional intelligence. The entire team shared their preliminary understanding of the data through regular discussions; (2) Generating preliminary codes: SJJ used NVivo 12.0 to perform open-coding on the full-text data. Codes were grouped based on similarities and differences, forming a working framework of codes within categories. (3) Generating preliminary themes: Three researchers (SJJ, FXH, ZLH) collaboratively consolidated similar codes to form candidate themes. During this process, the team repeatedly compared the relationships among codes, identified clusters of codes exhibiting developmental characteristics, and preliminarily synthesized them into three potential themes.; (4) Review and development of themes: The categories in the framework were mapped into a matrix: codes for each category were listed in separate columns, participants were listed in rows, and the original data for each participant under the corresponding code were summarized in the matrix cells. Three researchers (SJJ, FXH, ZLH) collaborated on this stage, cross-referencing the matrix to verify the consistency between codes and raw data, and repeatedly comparing and adjusting the thematic structure; (5) Defining and naming themes: Based on the matrix, SJJ systematically organized the relationships among codes and the intrinsic connections between codes and participant narratives, identified an integrated thematic structure, and clearly defined and named the connotations of each theme. (6) Drafting the report and manuscript: A systematic interpretation was developed around the final themes, and research findings were presented in conjunction with interview data to complete the drafting of the research report and manuscript. Data analysis was conducted using NVivo 12.0 software. The relevant analysis process is presented in Table 2.

**Table 2:** Example of the data analysis process.

Transcript	Researcher’s text	Coding	Category	SubCategory
“The biggest help is that it quickly gathers information for us, saving us the time it would take to compile it ourselves.” (N4)	Initially, students are strongly motivated by performance expectations and view AI as a tool for improving learning efficiency rather than a partner in deep learning. This drive for efficiency serves as the starting point for technology adoption, but the mere pursuit of efficiency does not automatically translate into the development of emotional intelligence.	Quickly gather information and save time	From Efficiency-Driven to Empathetic and Targeted	Efficiency-driven
“If you want to go deeper, you need to keep fine-tuning it with him. If I do not express myself clearly enough, the images he generates will not meet my expectations.” (N2)	When faced with obstacles related to their expectations, students are compelled to articulate their needs more precisely. This process of technical adjustment becomes a key factor in fostering deep empathy.	Further coordination is needed; instructions are not precise.	From Efficiency-Driven to Precise Empathy	Technical barriers
“My classmates and teachers are very supportive of using AI.” (N1)	The shift in attitudes among teachers and peers—from opposition to support—provides students with a sense of psychological safety and lowers the psychological barrier to attempting AI-based emotional communication exercises. Social influence not only motivates usage behavior but also creates the conditions for the internalization of social skills.	Support from teachers and students fosters a positive atmosphere	From Mimicking AI to Independent Expression	Social Impact
“The school offers a virtual simulation experiment on diabetic foot.” (N1)	The enabling conditions provided by the school, such as virtual simulation resources, have created a basic AI learning environment for students. However, students generally report a lack of systematic guidance tailored to nursing scenarios, indicating that these enabling conditions still need to be strengthened.	The school provides virtual simulation resources	From Technical Setbacks to Mental Rehearsals	External Support
“At the time, the patient was in the late stages of lung cancer, and I was a nursing intern.” (N1)	Senior students are able to translate the situational knowledge gained from clinical placements into role-playing scenarios in AI simulations, demonstrating the integration of technical knowledge with nursing content. This ability to construct scenarios is key to refining empathy from a vague to a precise understanding.	Set up patient roles for simulation	From Efficiency-Driven to Empathetic and Targeted	Scenario Building
“Have AI simulate a teacher and rehearse before communicating.” (N13)	By actively having the AI play the role of a teacher to practice communication, students demonstrate their commitment to the role and the application of learning strategies. This mental rehearsal helps them prepare emotionally before engaging in real-life interactions.	Rehearse the conversation with the teacher	From Mimicking AI to Independent Expression	Immersion
“We need to make corrections to the results and add our own input.” (N5)	Students are able to identify the limitations of AI-generated output and critically evaluate and adapt it. This ability is a crucial step in the process of internalizing social skills from external models into autonomous expression, and it also reflects the higher-order integration of TPACK.	Requires manual correction	From Mimicking AI to Independent Expression	Critical Integration
“Critically ill patients in the hematology department may seem compliant on the surface, but they are actually extremely anxious, and negative feedback can cause them to become very agitated.” (N11)	Drawing on their clinical experience, senior students are able to identify patients’ underlying, masked emotions. At this stage, empathy has evolved from emotional recognition into precise emotional understanding.	Recognizing a patient’s underlying anxiety	From Efficiency-Driven to Empathetic and Targeted	Precise Empathy
“If it were about my studies, and he always praised me, I might think there was something wrong with him, and it would actually make me reflect on myself.” (N13)	The AI’s overly positive feedback triggered self-doubt in the students, and this sense of unreality actually became a key catalyst for self-reflection.	Reflecting on oneself through AI feedback	From Technical Setbacks to Mental Rehearsals	Self-awareness
“When you know what to expect, you can act with confidence.” (N13)	Through repeated AI-simulated exercises, students have developed the ability to mentally rehearse scenarios, enabling them to handle real clinical situations with greater composure. This reflects the development of self-management skills and is also a key indicator of the formation of intrinsic motivation.	I have a clear idea of what to expect and feel more confident	From Technical Setbacks to Mental Rehearsals	Self-management
“The biggest benefit is definitely the increased efficiency; I was able to quickly identify the right direction for my studies.” (N9)	Efficiency (performance expectations) is the strongest motivator for students to use AI. However, as they accumulate successful experiences, their motivation gradually shifts from external rewards to internal psychological readiness and professional confidence.	Efficiency Drives Consistent Practice	From Technical Setbacks to Mental Rehearsals	Self-motivation
“AI will definitely change the way I speak, because the way I comforted people back then just wasn’t working.” (N1)	Students acknowledged that AI had influenced their communication strategies and proactively made adjustments. This process reflects a shift in social skills from imitation to autonomous expression, with critical integration playing a key role in this transition.	AI influences the way we speak and shapes our communication strategies	From Mimicking AI to Independent Expression	Expression Adjustment
“AI training can be partially applied in clinical settings, but because there is significant variation among individuals, AI is unreliable.” (N1)	Students are keenly aware of the gap between AI simulations and clinical reality, but rather than abandoning their use, they have adopted a cautious approach of using them as a reference without blindly following them. This tension serves as a resource for cultivating critical judgment.	There is a gap between AI and clinical reality	From Mimicking AI to Independent Expression	Simulation gap
“Concerned about privacy breaches, as AI may store chat logs.” (N13)	Concerns about privacy and ethics have not entirely deterred students from using AI; rather, they have prompted them to develop more responsible usage habits. This ability to set boundaries is, in itself, a manifestation of self-management skills.	Concerns about privacy breaches	From Technical Setbacks to Mental Rehearsals	Privacy Management
“I hope the instructor will relate AI more closely to specific scenarios in nursing practice.” (N2)	Students expect guidance on using AI in nursing-specific scenarios, rather than general technical training. This provides educators with a clear direction: they should integrate AI literacy training into professional contexts.	Looking forward to seeing it in real-world nursing settings	Other	Recommendations for Curriculum Integration

## 3 Results

### 3.1 Participant information

This study included 19 undergraduate nursing students. All participants met the inclusion criteria: they were nursing majors currently enrolled in their first, second, third, or fourth year of study, and had experience using generative AI tools (such as DeepSeek, Wenxin Yiyan, and Doubao) to complete academic assignments. The sample included 16 female students (84.2%) and 3 male students (15.8%). The distribution by academic year was as follows: 5 first-year students (26.3%), 5 second-year students (26.3%), 5 third-year students (26.3%), and 4 fourth-year students (21.1%). Regarding clinical experience, all third- and fourth-year students had completed clinical rotations, while some first- and second-year students had experience with hospital volunteer work or internships. Participants reported using a variety of GenAI tools, with Doubao being the most frequently mentioned (*n* = 17), followed by DeepSeek (*n* = 11) and Wenxin Yiyan (*n* = 4). In terms of frequency of use, 11 participants reported using the service daily, while 8 reported using it several times a week. The purposes of use included academic support (such as doing homework, researching information, and practicing conversations), emotional support (such as emotional analysis, emotional counseling, and advice on minor psychological issues), and daily life assistance (such as health consultations and looking up general lifestyle information). To protect privacy, all participants used pseudonyms. Participants’ basic information is shown in Table 3.

**Table 3:** Participant characteristics (*N* = 19).

Number	Grade	Gender	Use GenAI types	Frequency of use	Purpose of use
N1	Class of 2023	Female	Doubao	Several times per week	Homework, innovation project
N2	Class of 2025	Female	Doubao	Daily	Homework, general knowledge queries, topics of interest
N3	Class of 2025	Male	Doubao	Several times per week	Emotional support, sentiment analysis, coursework
N4	Class of 2025	Female	Doubao, Deepseek	Daily	Information search, competitions, drawing
N5	Class of 2025	Male	Doubao	Several times per week	College forms, life queries, simulated dialogue
N6	Class of 2025	Female	Doubao, Wenxin Yiyan	Several times per week	Poster design, handwritten newsletters, simulated patient dialogue
N7	Class of 2022	Female	Deepseek, Wenxin Yiyan	Daily	Homework, information search, sentiment analysis
N8	Class of 2023	Female	Doubao, Wenxin Yiyan	Several times per week	Photo editing, avatar design, script writing
N9	Class of 2023	Female	Doubao, deepseek	Daily	Study assistance, scenario drama, information search
N10	Class of 2023	Female	Doubao, deepseek	Several times per week	Terminology search, information search, simulated dialogue
N11	Class of 2022	Female	Doubao, deepseek	Daily	Emotional feedback, medication knowledge queries, image generation
N12	Class of 2022	Female	Doubao, deepseek	Daily	Homework, topics of interest, complex problem organization
N13	Class of 2023	Female	Doubao, deepseek, Wenxin Yiyan	Several times per week	Posting notices, information search, simulated teacher dialogue
N14	Class of 2024	Male	Doubao	Daily	Game guides, restaurant reviews, medication queries, patient emotion analysis
N15	Class of 2024	Female	Doubao, deepseek	Daily	Study questions, competition information search, patient emotion analysis
N16	Class of 2024	Female	Doubao	Daily	Difficult problem consultation, extracurricular practice
N17	Class of 2024	Female	Doubao, deepseek	Daily	Life queries, health consultation, study scenario simulation
N18	Class of 2024	Female	Doubao, deepseek	Several times per week	Life issues, auxiliary medical judgment, information search, simulated patients
N19	Class of 2022	Female	Deepseek	Daily	Study material search, thesis topic selection, psychological consultation

### 3.2 Categories and subcategories

In this study, the interview data were managed using NVivo 12.0 software and analyzed following the six-stage process of reflexive thematic analysis (RTA). Upon completion of the analysis, 238 initial codes were identified and synthesized into three core themes (and several subthemes). The correspondence between the two sets of codes is shown in Table 4.

**Table 4:** Total number of class codes in each category.

Categories	Subcategories	Frequency code
From efficiency-driven to empathetic and targeted	Efficiency-driven (performance expectations)	21
	Technical barriers (effort-expectancy)	17
	Scenario-building skills	19
	Precise empathy	13
From mimicking AI to independent expression	Social impact	16
	Role engagement and learning strategies	20
	Critical evaluation and adjustment	21
	Social skills	16
	The tension between simulation and reality	17
From technical setbacks to mental rehearsals	Enabling conditions (external support)	11
	Self-awareness	10
	Self-management	15
	Self-motivation	12
	Privacy and ethics management	12
Other	Recommendations for Curriculum Integration	18

In this study, coding frequency is used solely for descriptive purposes to illustrate the distribution of each subcategory within the data; it is not used as a basis for determining the importance or validity of themes. Within the framework of reflexive thematic analysis, the value of a theme lies in its analytical depth and explanatory power, rather than in the frequency with which it appears in the coding. Braun and Clarke (2022) (18) explicitly state that reflexive thematic analysis does not rely on coding frequency or consensus to establish the validity of themes; rather, it constructs the meaning of themes through the researcher’s deep engagement with the data and reflective interpretation.

This study’s findings reveal an overarching theme: differences in undergraduate nursing students’ experiences with emotion recognition, interactive expression, and self-regulation when assisted by generative AI. As shown in Figure 1, this theme comprises three interrelated subthemes that illustrate the typical characteristics of students’ experiences with generative AI across academic years. Theme 1, “From Efficiency-Driven to Precise Empathy,” focuses on differences in empathy experiences. Students across academic years described experiences ranging from being efficiency-driven and encountering technical barriers to drawing on clinical experience. Theme 2, “From Mimicking AI to Autonomous Expression,” focuses on internalising social skills. Students described varying experiences ranging from direct application, recognition, and adjustment to internalised expression, as well as a simulation gap that persisted throughout the process. Theme 3, “From Technical Frustration to Mental Rehearsal,” integrates experiences related to self-management, self-awareness, and self-motivation, including training in coping with difficulties, privacy management, AI feedback and self-awareness, and the transformation of intrinsic motivation. The three themes correspond to different dimensions of emotional intelligence: empathy, social skills, and the integration of self-management, self-awareness, and self-motivation. Students in different grade levels reported distinct characteristics of their experiences.

**Figure 1:**
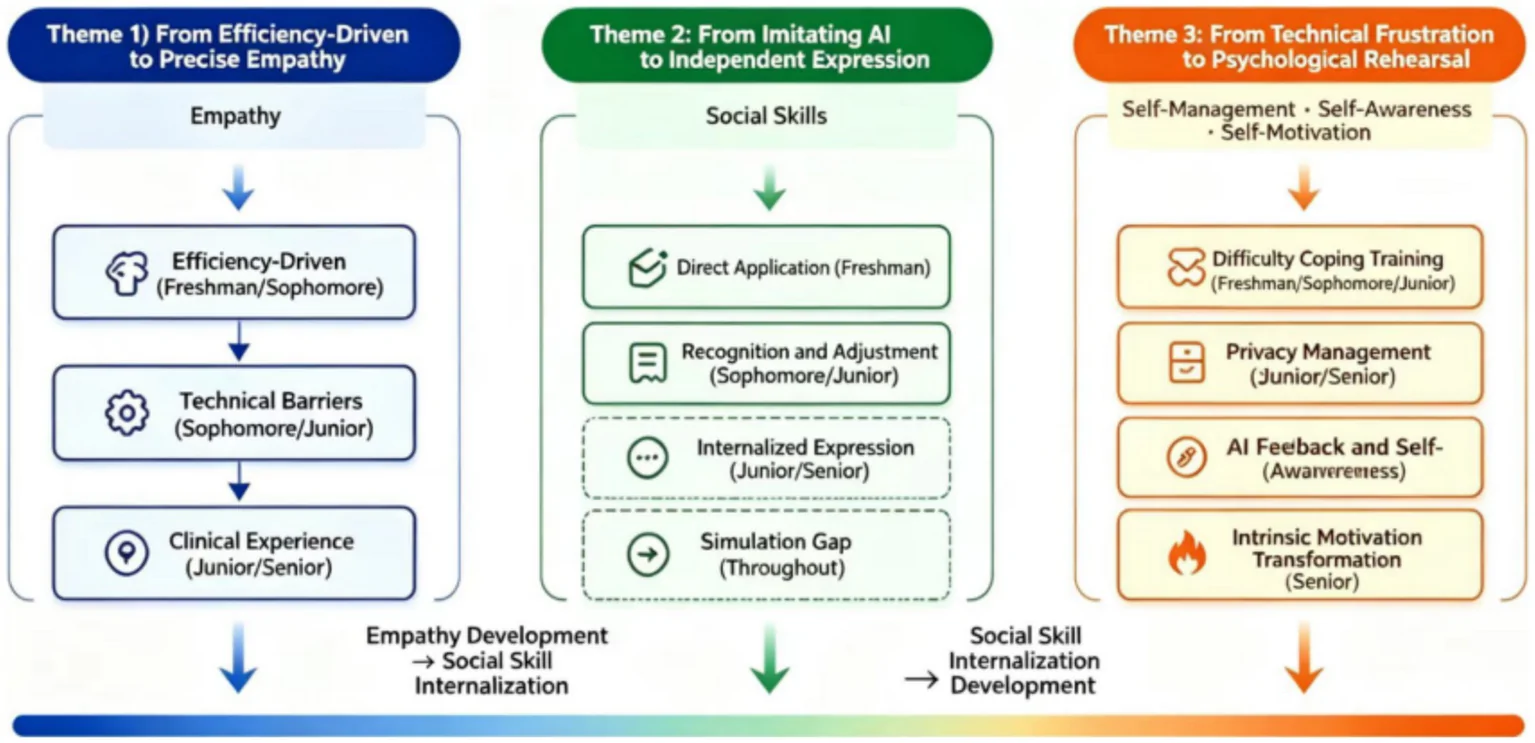
A model of cross-grade-level differences in the experiences of undergraduate nursing students using generative artificial intelligence for emotional intelligence learning.

#### 3.2.1 From efficiency-driven to precise empathy

For younger students, the primary motivation for initially using generative AI is to improve learning efficiency and complete tasks quickly. The simulated scenarios they create tend to be relatively simple and general, and their recognition of patients’ emotions remains limited to superficial labels. While this drive for efficiency serves as the starting point for technology adoption, the mere pursuit of efficiency does not automatically translate into the development of empathy. Reflecting on their freshman year, one student said, “The biggest benefit was increased efficiency, because many repetitive tasks could be completed more quickly, and by asking AI questions, I was able to quickly find a direction for my studies” (N9, junior). Another freshman described her scenario-setting process, noting, “I created a pregnant woman character who was feeling quite anxious, even though she is usually quite cheerful” (N6, freshman). At this stage, students view AI merely as a tool to improve learning efficiency, rather than as a partner for deep emotional understanding.

**Figure 2:**
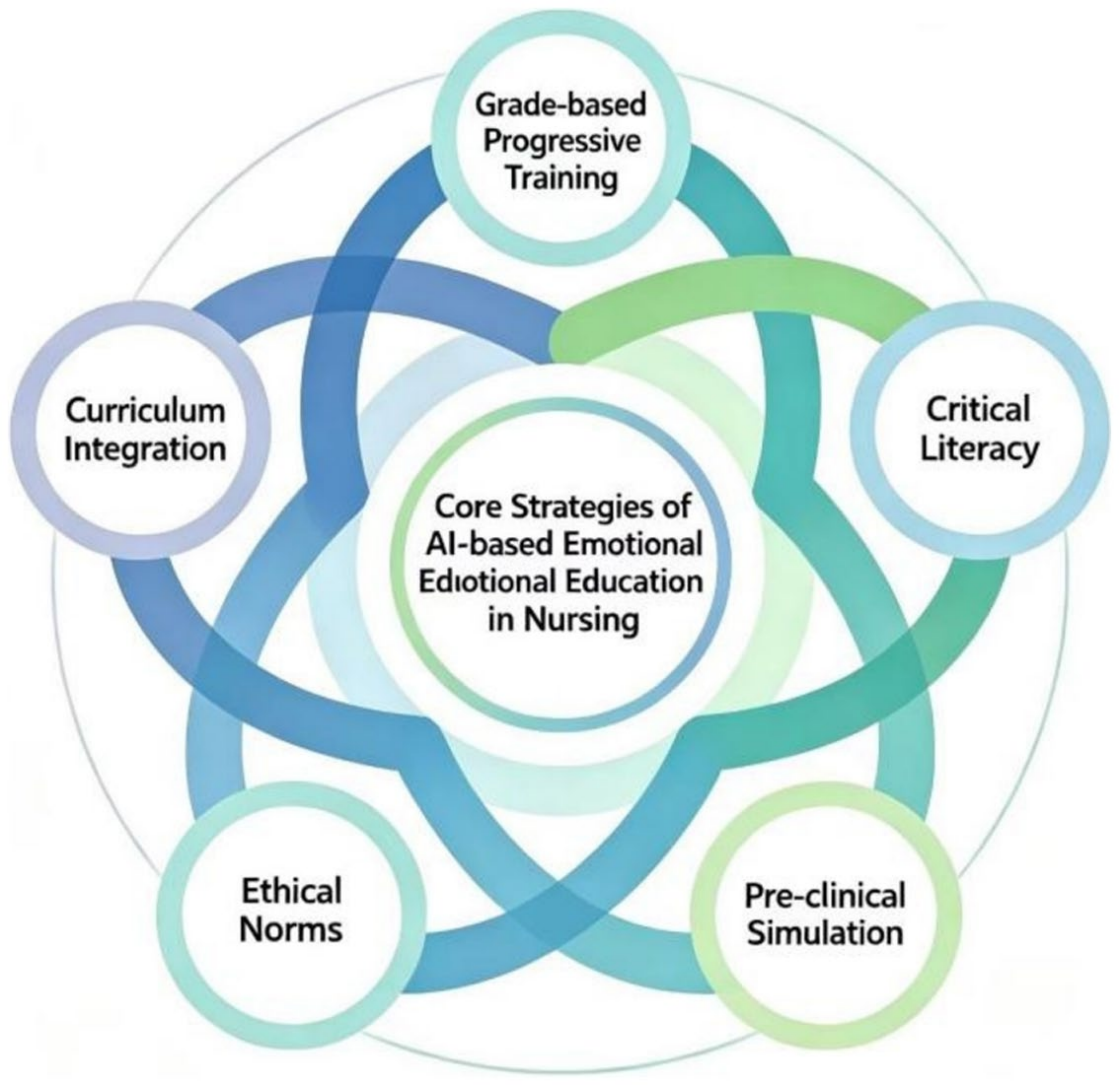
Exploratory educational implications for AI-assisted emotional intelligence development among undergraduate nursing students based on their perceptual experiences. This diagram centers on the “core strategies for AI-enhanced emotional education in nursing,” surrounded by five key educational measures: grade-level-specific training, curriculum integration, critical literacy, ethical standards, and pre-clinical simulation training. The five nodes are arranged in a circular pattern, illustrating the holistic framework in which these strategies mutually support and synergistically advance one another. The diagram is intended to serve as a visual reference for nursing educators seeking to systematically plan the integration of generative AI into emotional education.

However, when students attempt to simulate more realistic clinical communication, they commonly encounter difficulties in designing prompts and issues with AI responses that do not align with their input. These technical barriers prompted students to describe patients’ conditions, personalities, and emotional states in greater detail, and upperclassmen were more likely to describe uses of GenAI that incorporated clinical contextualization and critical reflection. A junior student commented, “The AI often doesn’t understand what I’m saying, and the answers it gives vary, so I don’t know which one is correct” (N9, junior). Another freshman also mentioned, “Because my verbal expressions aren’t precise enough, the AI’s responses often don’t meet my expectations” (N2, freshman). Researchers believe that this cognitive load prompts students to describe patients’ emotional states in greater detail.

As they gain more clinical internship experience, upperclassmen tend to construct more complex roles that incorporate factors such as the stage of the illness and the patient’s psychological state; their descriptions exhibit greater contextualization within clinical settings and critical reflection. One junior student described, “I created the role of a patient with advanced lung cancer and interacted with the patient as a nursing intern, reassuring the patient that the pain from the needle was normal, and there was no need to worry excessively, while sensing that the patient was in a state of fear” (N1, junior). Another senior student further noted, “Although patients in the hematology department appear compliant and responsive on the surface, they are actually very anxious inside; even the slightest negative feedback can cause them to become emotionally agitated” (N11, senior). Although students generally acknowledge that artificial intelligence cannot fully replace the emotional warmth found in real human interactions, through these exercises—which progress from vague to precise—they are more likely to describe emotional recognition experiences characterized by clinical contextualization and critical reflection. A junior student shared her insights from her clinical rotation: “I noticed a young child without a parent present who felt uneasy in the unfamiliar environment. The charge nurse paid special attention to the child, and I believed this was precisely an expression of caring for the child’s emotions” (N9, junior).

#### 3.2.2 From mimicking AI to independent expression

Lower-grade students tend to accept AI outputs and use them directly as correct answers. At this stage, students have not yet developed critical thinking skills; they view AI as an emotionless tool and fail to scrutinize or filter its outputs. A freshman admitted, “I don’t view AI as a person, so it doesn’t seem strange to me, and I don’t really pay much attention to it” (N4, freshman). Students merely mechanically copy AI’s responses and have not yet truly incorporated these communication patterns into their own expression habits.

As they gained more experience using the system, students noticed that the AI’s responses felt unrealistic or did not align with clinical practice, so they proactively adjusted their prompts. A junior student remarked, “The AI’s answers are quite formal, so I ask it to speak in a more conversational style” (N9, junior). Another senior student also mentioned, “When I ask the AI a question, it always gives very positive feedback first and then asks if I need help. This feels very unrealistic to me, so I give it new instructions and continue asking everyday questions” (N11, senior). Reflecting on the AI’s overly positive feedback, another junior said, “If the AI keeps affirming me, I actually start to suspect something is wrong; this sense of unreality prompts me to engage in self-reflection” (N13, junior). The shift in instructors’ attitudes has provided students with a sense of psychological safety. A junior recalled, “In the past, teachers might have told us not to use AI and to think for ourselves, but this semester, the instructors in every course have pointed out how advanced AI has become and have been very supportive of its use” (N1, junior). Researchers believe this stage marks the starting point for the transition of social skills from external models to internalised abilities.

Upper-level students can comprehensively evaluate the applicability of AI recommendations and tailor them to the specific characteristics of individual patients. At this point, AI is no longer merely a tool that provides standard answers, but rather a conversational partner that stimulates independent thinking. One senior student summarised, “I’ll generally adopt the AI’s style, but add my own words in certain areas” (N7, senior). Another student stated, “I’ll definitely ask the AI, but I’ll check whether the answer fits the patient’s situation. If the patient is outgoing, I’ll consider adjusting the tone; if it’s an older adults or a child, I’ll tweak the language to ensure the message is conveyed accurately” (N5, freshman).

Almost all students recognised the gap between AI simulations and real clinical situations, but this did not lead them to abandon its use; instead, it fostered a cautious approach to using AI as a reference without following it unthinkingly. Researchers believe this tension serves as a transitional resource for cultivating critical judgment. One senior student pointed out, “The biggest obstacle is that AI cannot analyse specific problems on a case-by-case basis; the answers it provides are relatively broad” (N19, senior). Another junior student also stated, “I don’t think AI works because humans are, after all, complex, while AI is just a machine. Even if it becomes more empathetic in the future, it still cannot compare to real-life interpersonal interactions” (N10, junior). Students clearly recognise the limitations of AI simulations and use them to make judgments and adjustments.

#### 3.2.3 From technical frustration to mental rehearsal

The challenges encountered when using generative AI provide students with a low-risk environment in which to practice managing frustration and anxiety. From the feelings of being overwhelmed and wanting to give up in the early grades to the repeated trial-and-error and perseverance in the later grades, students described experiences related to emotional regulation as they navigated technical difficulties. A senior student described the experience: “When I asked the AI to generate an image, it often produced a jumble of gibberish. I didn’t know how to give the right instructions, which was frustrating, but I kept trying until I got what I wanted” (N11, senior). This process of persisting and adjusting in the face of technical challenges is, in itself, training in self-management skills.

Privacy and ethical concerns have not completely deterred students from using AI; rather, they have prompted them to develop more responsible usage habits. This ability to set boundaries is, in itself, a manifestation of self-management skills. A sophomore said, “I’m concerned that AI might infringe on my privacy. For example, I once asked an AI how well it knew me, and it actually named the university I attend. Even when I clicked to start a new conversation, the AI still remembered my details. “So I’m careful not to ask anything too personal” (N17, sophomore). Another student expressed similar concerns: “My biggest worry is privacy breaches; if I ask for personal information, I’m afraid my privacy will be compromised” (N18, sophomore). Through their use of AI, students have gradually developed an awareness of privacy boundaries and learned to manage them proactively.

Some students use generative AI to analyse their emotions or gain insight into their issues by reflecting on the AI’s overly positive feedback. In this context, AI provides objective feedback, thereby fostering the development of self-awareness. A freshman remarked, “Sometimes after arguing with my girlfriend, there are things I can’t put into words—I don’t know why I feel that way—so I ask Doubao to analyse the cause” (N3, freshman). Another junior reflected, “If the AI keeps affirming me, I actually start to think there’s something wrong with it; that sense of unreality prompts me to reflect again” (N13, junior). The non-judgmental environment AI provides allows students to safely explore their emotional states.

Students’ motivation for using generative AI to practice emotional communication has shifted from external factors to intrinsic motivation. As students succeed repeatedly through practice, these positive experiences drive the shift from external to intrinsic motivation. One junior initially noted, “The biggest benefit is increased efficiency—many repetitive tasks can now be completed faster, and by asking the AI questions, I can quickly identify my learning direction” (N9, junior). Another junior stated, “I feel more relaxed and have a better sense of what to expect. Understanding the full range of possibilities—from the worst-case to the best-case scenario—gives me a sense of expectation, and I feel more confident in what I do” (N13, junior). A senior student said, “AI always gives me positive feedback. Even though I know it’s just reassurance, it really lifts my spirits. I think AI is useful because it makes abstract concepts concrete—for example, what wet rales sound like or what inspiratory dyspnea feels like—so they’re no longer so abstract” (N11, senior). Even without systematic guidance, students demonstrated prudent self-motivation. A freshman remarked, “Although AI is convenient, I feel I still need to correct the results and incorporate my own insights” (N5, freshman). This commitment to independent thinking and professional autonomy is itself part of their intrinsic motivation.

### 3.3 Connections between themes

The three themes correspond to students’ perceptual experiences across different dimensions of emotional intelligence. In the first theme, lower-grade students reported experiences driven more by efficiency; some middle-grade students described difficulties caused by technical barriers; and upper-grade students described identifying patients’ emotions, often drawing on clinical experience. In the second theme, students reported a range of experiences, from direct application and recognition to adjustment and autonomous expression. In the third theme, students described their perceptual experiences regarding emotional regulation, self-awareness, and intrinsic motivation. Students in different grade levels reported distinct characteristics of their experiences. First-year students’ descriptions largely focused on efficiency-driven and imitation phases, while second- and third-year students more often described overcoming obstacles. Fourth-year students, in turn, more often mentioned experiences involving the integrated application of clinical experience and critical thinking.

## 4 Discussion

Through in-depth interviews with 19 undergraduate nursing students, this study explored their experiences with using generative artificial intelligence (GenAI) for emotional learning, as well as the factors that facilitate this process. In contrast to the findings of previous reviews, which noted that “existing strategies tend to focus on empathy and social skills, with limited attention to self-awareness, self-management, and self-motivation” (17), this study presents the students’ reported experiences regarding emotion recognition, interactive expression, and self-regulation during their use of GenAI, as well as differences in these experiences across different academic years. The discussion below is organized around three core themes, each addressing a specific research question: Theme 1 addresses students’ perceptions of experiences related to empathy; Themes 2 and 3 address students’ perceptions of experiences related to social skills, self-management, self-awareness, and self-motivation; and the differences across grade levels observed in these three themes address the variations in students’ reported experiences. The correspondence between the three themes and Goleman’s five dimensions is as follows: Theme 1 focuses on empathy; Theme 2 focuses on social skills; and Theme 3 integrates the three dimensions of self-management, self-awareness, and self-motivation. Data analysis shows that when students describe their experiences with self-management, self-awareness, and self-motivation, these three elements often appear together in the same narrative; therefore, this study combines these three dimensions into Theme 3.

### 4.1 Moderate tension between performance expectations and effort expectations: a conditional factor in the experience of empathy

Research has found that performance expectations are the strongest driver of students’ use of GenAI, which is consistent with the Unified Theory of Acceptance and Use of Technology (UTAUT) (25). Students generally believe that using generative AI can improve learning efficiency, prompting them to engage in repeated simulation training (26). However, the relationship between the intensity of performance expectations and changes in emotional intelligence is not linear. An excessive pursuit of efficiency actually leads students to blindly adopt AI recommendations, thereby inhibiting the demonstration of critical integration skills. Students reported that when they perceived AI as useful and encountered moderate operational difficulties (such as challenges in designing prompts), they tended to describe patients’ emotional states in greater detail. This perceived association warrants further investigation, but this study cannot determine whether a causal relationship exists between the two. For students who are able to consistently follow adjustment instructions and accurately describe patients’ emotional states, they are, in fact, practicing the transformation of intangible emotional experiences into tangible nursing communication content. This reflects the tendency of upperclassmen to describe uses of GenAI that are characterized by clinical contextualization and critical reflection.

Previous research has demonstrated that performance expectancy is a significant predictor of nursing students’ intention to use GenAI (27, 28). This study found that when applying the UTAUT theory to the development of emotional intelligence, the key lies in maintaining a moderate balance between performance expectancy and effort expectancy. Students must both perceive AI as useful (performance expectations) and encounter an appropriate level of operational difficulty (effort expectations); the combined effect of these two factors helps students describe deeper experiences of empathy, rather than any single factor being more effective the stronger it is. Shen et al. noted that students commonly face difficulties, such as incomplete responses and limited emotional understanding from AI, when using it (29). Some students describe technical obstacles as opportunities to practice patience and emotional regulation. This perception suggests that, under certain conditions, technical glitches may be experienced by students as practice scenarios; however, this does not mean that technical obstacles themselves necessarily promote the development of emotional regulation skills. Educators should design tasks that require students to provide detailed descriptions of clinical scenarios, rather than striving for a completely obstacle-free operational experience.

From the perspective of integrating technological and content knowledge, upper-level students can construct complex characters that incorporate multidimensional information—such as medical conditions, emotions, and personality traits—based on the content knowledge they have accumulated through clinical internships. This is a clear manifestation of integrating technological knowledge and content knowledge within the TPACK framework (26). This finding suggests that the differences in experiences reported by students in different grade levels may be related to their accumulation of knowledge about clinical contexts, and it also suggests that educators should pay attention to the role of knowledge about clinical contexts in the integration of technology. The more complex scenario-construction abilities demonstrated by upper-level students may stem in part from real-world patient interaction experiences accumulated during clinical rotations, rather than being entirely attributable to GenAI use. Because this study used a cross-sectional design, it could not disentangle the independent contributions of clinical experience and GenAI use to scenario-construction abilities. Future research could explore this issue further by adopting a longitudinal design or controlling for clinical experience.

Furthermore, it is important to distinguish that while students improved the accuracy of their descriptions of patient emotions with GenAI assistance—that is, they were able to identify and describe patients’ emotional states more specifically (e.g., distinguishing between fear, anxiety, and unease)—this does not equate to deep empathy. Theoretically, empathy is a multidimensional construct comprising two core components: cognitive empathy (perspective-taking) and affective empathy (emotional resonance) (30). The emotional recognition skills students develop with GenAI assistance primarily fall at the cognitive level. In contrast, deep empathy involves understanding patients’ subjective experiences and emotional resonance, which require authentic interpersonal interactions and accumulated clinical experience. A study on GenAI-enhanced VR simulations also noted that current technology faces “difficulties in establishing genuine empathy with virtual agents, highlighting the critical gap between cognitive training and emotional resonance” (31). GenAI simulations can give students opportunities to practice describing emotions, but deeper empathy development still requires real clinical settings.

### 4.2 Critical evaluation and adaptability: key factors in the internalization of social skills

This study found that social skills are not automatically internalised; the key factor is the ability to critically evaluate and adjust one’s behavior. Lower-grade students tend to apply AI-generated responses directly, whereas upper-grade students can identify the artificiality or clinical inaccuracies in AI responses and transform external models into their own competencies by adjusting instructions, selectively adopting content, and personalizing information. This process reflects the synergistic interaction between social influence and critical integration.

Specifically, the shift in attitude among theory instructors—from discouraging the use of AI to actively recommending it—combined with the demonstrations provided by clinical instructors during practical training, reinforced students’ willingness to use the technology. Meanwhile, patients’ and their families’ reactions during real-world interactions directly motivated students to adjust their communication strategies. These social influences lowered the psychological barrier to students’ willingness to try generative AI and strengthened their intrinsic motivation to actively practice emotional communication (32).

This finding aligns with the UTAUT framework, which posits that social influence indirectly drives technology use by shaping students’ behavioral intentions (26). Furthermore, some students reported that support from teachers and peers influenced their willingness to continue using AI, and that they gradually developed their own ways of expressing themselves through repeated practice. This report reflects the students’ perceptions rather than an objective validation of the process of internalizing social skills. Lower-grade students reported that when they first used generative AI, most of them directly adopted the answers provided by the AI; upper-grade students, on the other hand, reported that through their interactions with AI, they gradually developed their own criteria for judgment, no longer simply parroting the AI’s words but instead expressing themselves in their own way (33).

The process described by the students—from imitation to adaptation to independent expression—suggests that experiences related to social skills may involve the critical evaluation and selective adoption of AI outputs; however, the mechanisms underlying this process require further research to verify. By comparing AI suggestions with real clinical settings, the students learned to distinguish between perfunctory consolation and genuine empathy. Research indicates that learners achieve better outcomes when they first compare different case studies and then learn the underlying principles, rather than simply listening to lectures (34). This represents a crucial shift in the development of social skills—from rote memorization to autonomous learning (32). Research has found that the core value of LLM-based virtual patient simulations lies in providing a safe, repeatable clinical practice environment (12), while AI avatar technology promotes student self-reflection through timely feedback (35). At the same time, AI simulations offer a valuable preparatory setting for students lacking clinical experience (11). Students learn to discern and make judgments within these simulated scenarios, practice repeatedly in a safe environment, and adjust their strategies based on immediate feedback. Therefore, AI simulations are not substitutes for real clinical settings, but rather transitional training tools that help students build foundational judgment skills and prepare them for real clinical interactions (36).

### 4.3 Overcoming technical barriers and ethical concerns: the role of training in self-management skills

This study found that the impact of technical barriers and ethical concerns on self-management skills is twofold. The UTAUT framework typically views effort expectations (difficulty of use) and facilitating conditions (external support) as factors that either promote or hinder technology acceptance. In this study, some students reported that technical difficulties (such as repeated failures with prompts) led to feelings of frustration, but they also described these challenges as opportunities to practice emotional regulation. Privacy and ethical concerns may inhibit use, yet they also prompt students to develop responsible use habits and an awareness of boundaries, which, in itself, constitutes a manifestation of self-management skills (29, 37).

Students’ reports of their experiences with self-management exhibited nonlinear patterns. Some students mentioned that GenAI’s overly enthusiastic feedback made it feel unrealistic, while difficult prompts and technical obstacles led to feelings of frustration. These students described these negative emotions as opportunities to practice emotional regulation. Through repeated refinement of their instructions and adaptation to the AI, students learned to manage anxiety and persevere. In AI simulations, students can safely experience the full cycle of failure, emotional fluctuations, adjustment, and improvement, and can repeat this process an unlimited number of times. Research indicates that AI-assisted pre-simulation guidance can significantly enhance students’ psychological readiness and self-confidence while providing a safe practice environment (38, 39). More importantly, some students mentioned that AI’s insincere feedback led them to reflect on genuine empathy and to pay closer attention to their own emotional responses. This shift in perception is noteworthy, but it does not yet equate to the development of self-monitoring skills. From an educational psychology perspective, this constitutes a metacognitive monitoring practice. Students not only learn how to respond to patients but also how to observe and regulate their own emotional reactions under stress. Existing models have highlighted the interplay between metacognition, motivation, and emotion in self-regulated learning (40). However, overreliance on GenAI may undermine self-reflection and critical thinking skills (10). Therefore, the technical challenges in AI simulations should not be viewed as obstacles but rather as a crucial component in training emotional regulation skills.

From the perspective of emotional intelligence theory, this finding fills a gap identified in previous reviews (17). There is very little existing research on instructional strategies for self-management. The learning experiences, technical difficulties, and ethical concerns reported by students may be perceived by them as opportunities to practice self-regulation, but this does not mean that these factors in and of themselves necessarily promote the development of self-regulation skills. Education on privacy protection should emphasize proactive boundary management, guiding students to internalize ethical awareness as a capacity for self-regulation rather than simply prohibiting use (37).

### 4.4 A safe environment and positive experiences: factors that promote self-awareness and self-motivation

Previous research has paid little attention to the experiential characteristics of self-awareness and self-motivation, and this study provides preliminary evidence based on students’ reports of their experiences (17). Some students reported proactively using AI to analyze their emotions or becoming aware of their issues through the AI’s overly positive feedback. These students described how the AI provided a safe, non-judgmental, pressure-free environment that let them express their inner confusion without fear of judgment. Research indicates that AI and augmented reality technologies can serve as emotional triggers and mirrors for reflection, allowing learners to practice therapeutic communication and cultivate self-awareness and emotional regulation skills (41). Other studies also note that a core objective of AI avatars combined with gamified instruction is to foster self-awareness and emotional regulation skills among nursing students (39). AI-driven structured reflection can help students analyze clinical interactions systematically, thereby fostering self-awareness and self-regulation skills (42). However, this study could not verify whether these reported experiences translated into tangible changes in self-awareness.

Regarding self-motivation, some students reported perceiving a gradual shift in their motivation from external factors (such as efficiency or instructor recommendations) toward intrinsic motivation. They attributed this shift to repeated success through practice. However, without sufficient external support, some students also described a cautious approach: using AI without allowing it to replace their own efforts. This suggests that students’ commitment to independent thinking and autonomous completion of professional tasks may be an important aspect of their self-motivation; however, this study could not determine whether a causal relationship exists between the two. Educators may help students develop a balanced understanding of the relationship between technology and professional growth by presenting real-world examples that demonstrate AI as a supportive tool rather than a substitute.

### 4.5 Implications for nursing education practice

Based on the above discussion, this study offers the following exploratory educational insights for nursing educators to consider. These recommendations are derived from the perceptions and experiences of 19 undergraduate nursing students and are intended as exploratory educational insights rather than evidence-based intervention strategies. The applicability of these recommendations requires further validation in studies involving larger samples and multiple institutions:

First, establish a grade-based system for cultivating technical literacy. The ability to integrate technology is closely linked to the accumulation of professional knowledge, suggesting that educators need to design differentiated training programs based on students’ developmental stages. For lower-year students, the focus should be on foundational operations and scenario-based training to help them establish a basic understanding of GenAI tools; for upper-year students, the emphasis should be on developing critical integration and clinical translation skills, guiding them to organically integrate AI simulations with clinical practice (43).

Second, systematically integrate GenAI tools into professional curricula. Situated learning theory emphasizes that the development of knowledge and skills should be embedded in authentic learning contexts (44). Students generally seek guidance on using AI in nursing-specific scenarios rather than generic technical training. Research has confirmed that integrating GenAI tools, such as ChatGPT, into nursing process training can significantly enhance nursing students’ problem-solving abilities, positive attitudes toward AI, and competencies in the nursing process (11). Therefore, GenAI-assisted tasks can be designed for courses such as “Nursing Communication” and “Fundamentals of Nursing,” allowing students to learn and reflect on the use of technology in professional contexts. Implementing the aforementioned concepts of grade-level development and curriculum integration in specific teaching scenarios can yield scenario-based intervention measures, as shown in Table 5, Figure 2.

**Table 5:** Implications for exploratory education based on students’ perceived experiences.

Application scenarios	Key dimensions of emotional intelligence	Specific teaching measures	Related research findings
Theoretical courses (such as “nursing communication”)	Empathy, social skills	Design a “human-vs.-AI” task: Students first manually write communication scripts, then generate them using AI, and compare the differences; teachers facilitate group discussions to guide students in distinguishing between superficial reassurance and genuine empathy in the AI’s suggestions (47)	A healthy balance between performance expectations and effort expectations; the ability to critically synthesize information
Practical course (on-campus simulation training)	Social skills, self-management	Set up an AI-simulated failure scenario: intentionally have the AI generate incorrect or incomplete information, and ask students to independently adjust the prompt while recording their emotional responses, thereby training their ability to manage frustration (39).	Turning technical challenges into self-management training resources
Before the clinical internship (mental rehearsal)	Empathy, self-management	Students are required to independently create AI patient profiles (including medical conditions, personality traits, and emotional states) based on cases from their clinical rotation departments and to conduct simulated dialogues. After the rotation, they compare these profiles with actual patient responses and write a reflective report (48).	Scenario-building skills (integration of TK and CK); using simulation gaps as transitional resources
During clinical rotations (ward interactions)	Social skills, self-awareness, self-motivation	Encourage students to use AI to rehearse communication scenarios: Before entering a patient’s room, use AI to simulate potential emotional situations; after the clinical rotation, engage in anonymous conversations with AI to process their emotions and maintain an emotional journal.	A safe environment fosters self-awareness; experiences of success drive self-motivation
Independent study after class	Self-awareness, self-motivation	Provide AI-powered emotional analysis tools to guide students in regularly reflecting on their emotions; organize sessions for sharing success stories, allowing students to discuss real-life examples of how AI has helped them improve their emotional intelligence (49).	A Safe Environment and the accumulation of positive experiences

Third, fostering critical technological literacy rather than tool dependency. Many students have expressed concerns about overreliance on AI; educators should guide students to view AI as a tool that supplements rather than replaces human judgment. Research clearly indicates that integrating AI into nursing education requires vigilance to prevent students from becoming overly reliant on AI tools, which could undermine their critical thinking and independent problem-solving abilities (11). The core of technology literacy education lies in cultivating students’ ability to balance technology-assisted learning with independent thinking. This means that educators must not only teach students how to use AI but also equip them to judge when to use it and when it is not appropriate.

Fourth, strengthen education on ethical standards for technology use. In response to students’ privacy concerns, schools should establish clear guidelines for technology use and incorporate privacy protection and ethical standards into the curriculum. Educators should not only inform students of the rules but also cultivate their ethical awareness, enabling them to make responsible judgments in specific situations. At the same time, educators need to guide students in carefully evaluating the accuracy of AI outputs and handling data involving patient information and personal privacy with due care (29).

Fifth, develop technology-assisted preclinical simulation training. Simulation-based teaching is a key method in nursing education (38). By leveraging AI to construct diverse clinical communication scenarios, we can provide students with a risk-free, repeatable practice environment, helping them build foundational skills and psychological preparedness before entering real clinical settings. At the same time, educators should guide students to clearly recognize the differences between AI simulations and clinical practice, treating these differences as learning resources themselves, and using comparisons between AI simulations and clinical practice to enhance future nursing education models (45).

### 4.6 Limitations and future directions

This study has certain limitations. First, the sample was drawn from a single institution and consisted primarily of female students (84.2%), which may limit the generalizability of the findings. Future research could include samples from multiple regions and institutions to explore differences in students’ AI usage across various educational contexts. Second, the study used a cross-sectional design, interviewing students from different grade levels at the same point in time. While this design can reveal differences in experiences among students in different grade levels, it cannot determine whether these differences stem from advancing grade levels, accumulated clinical experience, or variations in students’ exposure to AI across different admission cohorts (as discussed in Section 2.2, students in the Class of 2025 and earlier cohorts experienced digital transformation in different contexts). Therefore, the inter-grade differences presented in this study should be understood as cross-sectional observations rather than a longitudinal developmental sequence. Future research could use a longitudinal design to track changes in the same cohort of students over time, further validating this study’s findings. Third, the study relied on student self-reports, which may be subject to social desirability bias. Future research could integrate multiple data sources, such as behavioral observations and communication skills assessments, to enhance the objectivity of the conclusions. Fourth, this study focused on the student perspective; future research could incorporate teachers’ views on AI-assisted emotional teaching to more comprehensively reveal the pathways for integrating AI into emotional education in nursing (46).

## 5 Conclusion

This study explored Chinese undergraduate nursing students’ perceived experiences using generative artificial intelligence (AI) for emotional learning, as well as the factors influencing these experiences. Based on Goleman’s five-dimensional model of emotional intelligence and integrating the Unified Theory of Technology Acceptance and Use with the knowledge framework for integrating technology into disciplinary instruction, the study found that performance expectations were the primary motivation for students’ use of AI, and that a moderate balance between perceived usefulness and operational difficulty was associated with students’ ability to describe patients’ emotions in greater detail. Social influence affected students’ willingness to use AI, and repeated practice gradually prompted them to develop their own modes of expression. Some students viewed technical barriers as opportunities to practice emotional regulation, while privacy concerns led them to adopt more responsible usage habits. The non-judgmental environment AI provided encouraged emotional exploration, and positive experiences influenced students’ willingness to continue using it. Upperclassmen reported more experiences of integrating clinical context knowledge into their use of AI. Based on these exploratory findings, this study proposes several preliminary implications—including grade-level-specific support, curriculum exploration, critical technological literacy, and ethics education—for future research and practice. These implications require further validation in larger-sample, multi-institution studies.

## Data Availability

The raw data supporting the conclusions of this article will be made available by the authors, without undue reservation.
